# Supplementary material for: Noninvasive Brain Stimulation for Improving Cognitive Deficits and Clinical Symptoms in Attention-Deficit/Hyperactivity Disorder: A Systematic Review and Meta-Analysis
Source: Brain Sci. 2024 Dec 9;14(12):1237. doi: 10.3390/brainsci14121237 (PMC11674686; doi:10.3390/brainsci14121237)
Supplement: Supplementary file 1 [file brainsci-14-01237-s001.zip › brainsci-3345253-supplementary.pdf]

Supporting Information

According to the Cochrane Risk of Bias Tool, we assessed every study to low, high, unclear risks in selection, performance, detection, attrition, reporting, and other bias domain. Selection bias included two parts, namely random sequence generation (selection bias in table S1) and allocation concealment (selection bias_1 in table S1).

Selection bias refers to systematic differences in characteristics between groups being compared, which can result from inadequate randomization, allocation concealment, or participant recruitment processes. Furthermore, performance bias and detection bias are both related to the blinding of healthcare providers, participants, research personnel, and outcome assessors in a study, and can influence the results of the trial if not appropriately controlled. Then, attrition bias relates to systematic differences in missing data or withdrawals from the study, which may introduce bias if the reasons for attrition are related to the participants or outcomes being measured. Moreover, reporting bias involves selective reporting of outcomes based on their significance or direction, resulting in incomplete or biased representation of the study results. Additionally, the tool includes a category for "other bias," which encompasses any potential sources of bias not covered by the previous categories but that could still impact the study's validity. We have provided the reasons for each study's risk of bias assessment in Table S1.

Table S1. Risk of bias ratings with supporting evidence. Italics parts are the reasons for judgment.

**Studies**	**Selection Bias**	**Selection Bias_1**	**Performance Bias**	**Detection Bias**	**Attrition Bias**	**Reporting Bias**	**Other bias**
**tDCS studies**							
Cosmo 2015	Low	Low	Unclear<br>*Not described*	Low	Unclear<br>*Not described*	Low	Low
Breitling 2016	Low	Unclear<br>*Single-blind*	Unclear<br>*Not described*	Low	Low	High<br>*Flanker effect n/r*	Unclear<br>*one-tailed t-tests*
Cachoeira	Low	Low	Unclear	Low	Low	Low	Low

2017			*Not described*				
Soff 2017	Low	Low	Unclear<br>*Not described*	Unclear<br>*no blind effect*	Low	Low	Low
Allenby 2018	Low	Low	Low	High<br>*blinding failed*	Low	Low	Low
Jacoby 2018	Low	Low	Unclear<br>*Not described*	Low	Low	Low	Low
Soltaninejad 2019	Low	Unclear<br>*Single-blind*	Unclear<br>*Not describe*	Low	Low	High<br>*Stoop result n/r*	Low
Breitling 2020	High<br>*Pseudoran-dom*	Low	Unclear<br>*Not described*	Unclear<br>*no blind effect*	Low	Low	Unclear<br>*Turned down the subject's current*
Nejati 2020	Low	Low	Unclear<br>*Not described*	Low	Unclear	High<br>*Stroop effect n/r*	Unclear<br>*post-hoc tests*
Salehinejad 2020	Low	Unclear<br>*Single-blind*	Low	Unclear<br>*Experimenter knew allocation*	Low	Low	Low
Breitling-Ziegler 2021	Low	Low	Unclear<br>*Not described*	Low	Low	Low	High<br>*Baseline difference*
Nejati 2021	Low	Unclear<br>*Single-blind*	Unclear<br>*Not described*	Unclear<br>*Blind effect n/a*	Low	Low	Low
Barham 2022	Low	Low	Unclear<br>*Not described*	Unclear<br>*Blind effect n/a*	Low	Low	Low
Leffa 2022	Low	Low	Low	Low	Low	Low	Low
Nejati 2022	Low	Unclear<br>*Single-blind*	Unclear<br>*Not described*	Low	Low	Low	Low
Salehinejad 2022	Low	Unclear<br>*Single-blind*	Unclear<br>*Not described*	Unclear<br>*Blind effect n/a*	Low	Low	Low
D'Aiello 2023	Low	Low	Unclear<br>*Not described*	Unclear<br>*Blind effect n/a*	Low	Low	Unclear<br>*washout time is short*
Guimarães 2024	Low	Low	Low	Low	Low	Low	Low
rTMS studies							
Paz 2018	Unclear<br>*Not described*	Low	Unclear<br>*Not described*	Unclear<br>*Blind effect n/a*	Low	Low	Low
Alyagon 2020	Low	Unclear<br>*Single-blind*	Low	Unclear<br>*Blind effect n/a*	Low	Low	Low
Bleich-Cohen 2021	Low	Low	Low	Unclear<br>*Blind effect n/a*	Low	Low	High<br>*Baseline difference in ASRS scale*

tACS and tRNS studies							
Dallmer-Zerbe 2020	Low	Low	Low	Low	Low	Low	Unclear *Demographics n/a*
Nejati 2023	Low	Unclear *Single-blind*	Low	Unclear *Blind effect n/a*	Low	Low	Low
Kannen 2024	Low	Unclear *Single-blind*	Low	Low	Low	Low	Unclear *washout time is short*
Berger 2021	Unclear *Not described*	Low	Low	Unclear *Blind effect n/a*	Low	Low	Low
Dakwar-Kawar 2022	Unclear *Not described*	Low	Low	Unclear *Blind effect n/a*	Low	Low	Low
Dakwar-Kawar 2023	Low	Low	Low	Low	Low	Low	Low
Nejati 2024	Unclear *Not described*	Unclear *Single-blind*	Unclear *Not described*	Low	Low	Low	Low

The purpose of leave-one-out sensitivity analysis in meta-analysis is to assess the robustness and reliability of the pooled effect size estimate. It involves systematically leaving out one study at a time and recalculating the effect size estimate to evaluate how much each study influences the overall result. This analysis helps to identify influential studies that may affect the conclusion of the meta-analysis and to examine the stability of the results. The results of sensitivity analysis are showed in Table S2.

Table S2. The results of sensitivity analysis in tDCS studies.

**Domains**	**Studies excluded**	**Effect size**			**Heterogeneity**	
		SMD	95% CI	p value	I2	p value
**Inhibition**	Not exclude	-0.21	[-0.39, -0.04]	0.02	10%	0.33
	Cosmo 2015	-0.23	[-0.42, -0.04]	0.02	14%	0.29
	Breitling 2016	-0.22	[-0.40, -0.03]	0.02	16%	0.27
	Breitling 2016(1)	-0.22	[-0.40, -0.03]	0.02	16%	0.27
	Allenby 2018	-0.21	[-0.41, -0.02]	0.03	16%	0.27
	Soltaninejad 2019	-0.17	[-0.35, -0.00]	0.05	1%	0.44
	Soltaninejad 2019(1)	-0.23	[-0.42, -0.04]	0.02	15%	0.29

Inhibition	Nejati 2020	-0.22	[-0.41, -0.04]	0.02	15%	0.28
	Nejati 2020(1)	-0.19	[-0.35, -0.02]	0.03	0%	0.46
	Nejati 2020(2)	-0.2	[-0.38, -0.02]	0.03	13%	0.31
	Salehinejad 2020	-0.21	[-0.40, -0.03]	0.03	16%	0.27
	Breitling-Ziegler 2021	-0.24	[-0.41, -0.07]	0.006	3%	0.42
	Nejati 2021	-0.2	[-0.38, -0.02]	0.03	11%	0.33
	Nejati 2021 (1)	-0.25	[-0.42, -0.08]	0.004	0%	0.59
	Barham 2022	-0.18	[-0.35, -0.02]	0.03	0%	0.46
	Nejati 2022	-0.23	[-0.42, -0.03]	0.02	15%	0.28
	Salehinejad 2022	-0.22	[-0.41, -0.03]	0.02	16%	0.27
	D’Aiello 2023	-0.23	[-0.42, -0.04]	0.02	15%	0.28
Working memory	Not exclude	0.31	[0.03, 0.59]	0.03	48%	0.05
	Allenby 2018	0.33	[0.03, 0.64]	0.03	52%	0.02
	Breitling 2020	0.23	[0.01, 0.45]	0.04	18%	0.28
	Breitling 2020(1)	0.24	[-0.02, 0.50]	0.07	37%	0.11
	Nejati 2020	0.3	[-0.02, 0.61]	0.07	51%	0.03
	Nejati 2020 (1)	0.33	[0.02, 0.63]	0.03	52%	0.02
	Nejati 2020 (2)	0.31	[0.01, 0.61]	0.05	52%	0.02
	Breitling-Ziegler 2021	0.35	[0.07, 0.64]	0.02	47%	0.04
	Barham 2022	0.31	[0.00, 0.61]	0.05	52%	0.02
	Salehinejad 2022	0.35	[0.05, 0.65]	0.02	49%	0.03
	Nejati 2022	0.34	[0.03, 0.65]	0.03	51%	0.02
	D’Aiello 2023	0.3	[-0.01, 0.61]	0.06	52%	0.02
	Guimarães 2024	0.36	[0.08, 0.65]	0.01	45%	0.05
Cognitive flexibility	Not exclude	-0.58	[-1.71, 0.56]	0.32	84%	0.002
	Nejati 2020	-0.94	[-3.18, 1.30]	0.41	92%	0.0005
	Nejati 2020 (1)	0.07	[-0.39, 0.52]	0.77	0%	0.66
	Nejati 2020 (2)					
	Salehinejad 2022	-1.05	[-3.09, 0.99]	0.31	89%	0.003
Inattention	Not exclude	-0.66	[-1.33, 0.00]	0.05	84%	<0.00001
	Cachoeira 2017	-0.46	[-1.11, 0.20]	0.17	84%	<0.00001
	Soff 2017	-0.87	[-1.50, -0.24]	0.007	80%	0.0002
	Allenby 2018	-0.47	[-1.11, 0.18]	0.15	78%	0.0004
	Jacoby 2018	-0.77	[-1.53, -0.01]	0.05	85%	<0.00001
	Barham 2022	-0.7	[-1.46, -0.06]	0.07	87%	<0.00001
	Leffa 2022	-0.62	[-1.44, 0.20]	0.14	86%	<0.00001
	Nejati 2024	-0.77	[-1.52, 0.02]	0.05	86%	<0.00001
Hyperactivity/Impulsivity	Not exclude	-0.41	[-1.05, 0.23]	0.21	70%	0.02
	Cachoeira 2017	-0.16	[-0.61, 0.28]	0.48	38%	0.2
	Soff 2017	-0.66	[-1.33, 0.02]	0.06	63%	0.07
	Jacoby 2018	-0.44	[-1.39, 0.51]	0.36	79%	0.009
	Leffa 2022	-0.53	[-1.57, 0.51]	0.32	80%	0.007

Table S3. The results of sensitivity analysis in rTMS studies.

Domains	Studies excluded	Effect size			Heterogeneity	
		SMD	95% CI	p value	I2	p value
Core symptoms	Not exclude	0.04	[-0.48, 0.55]	0.89	54%	0.09
	Paz 2018	-0.06	[-0.73, 0.60]	0.85	66%	0.05
	Alyagon 2020	0.28	[-0.11, 0.66]	0.16	0	0.16
	Bleich-Cohen 2021	0.02	[-0.74, 0.77]	0.97	70%	0.97
	Bleich-Cohen 2021(1)	-0.11	[-0.75, 0.52]	0.72	57%	0.1

Subgroup analysis allows for a more nuanced understanding of the overall treatment effect. By examining the effect sizes within different subgroups, we gained insights into the potential moderating factors that might affect the outcomes of interest. This information can be useful for identifying specific populations or circumstances where the intervention is more or less effective. The results of subgroup analyses are showed below.

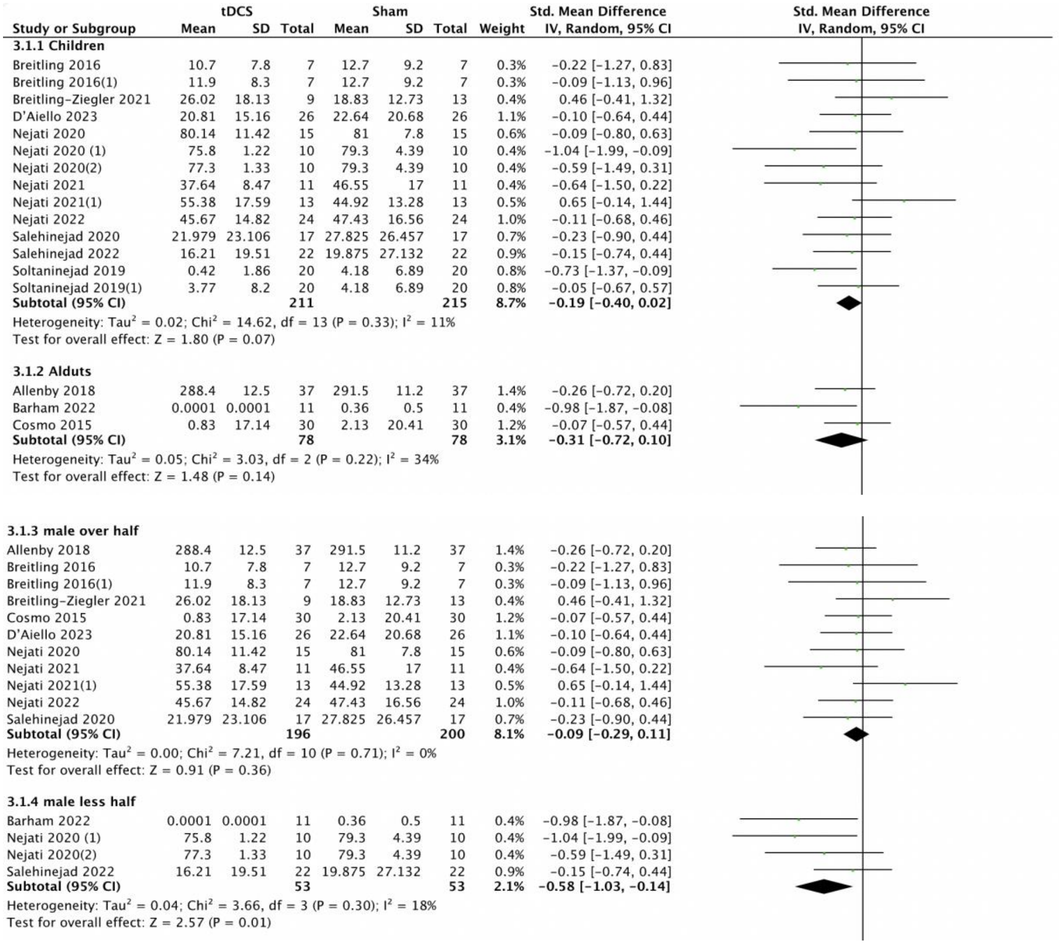

3.1.5 drug naive

Barham 2022	0.0001	0.0001	11	0.36	0.5	11	0.4%	−0.98 [−1.87, −0.08]
Breitling 2016	10.7	7.8	7	12.7	9.2	7	0.3%	−0.22 [−1.27, 0.83]
Breitling 2016(1)	11.9	8.3	7	12.7	9.2	7	0.3%	−0.09 [−1.13, 0.96]
Breitling–Ziegler 2021	26.02	18.13	9	18.83	12.73	13	0.4%	0.46 [−0.41, 1.32]
D'Aiello 2023	20.81	15.16	26	22.64	20.68	26	1.1%	−0.10 [−0.64, 0.44]
Nejati 2020	80.14	11.42	15	81	7.8	15	0.6%	−0.09 [−0.80, 0.63]
Nejati 2020 (1)	75.8	1.22	10	79.3	4.39	10	0.4%	−1.04 [−1.99, −0.09]
Nejati 2020(2)	77.3	1.33	10	79.3	4.39	10	0.4%	−0.59 [−1.49, 0.31]
Nejati 2021	37.64	8.47	11	46.55	17	11	0.4%	−0.64 [−1.50, 0.22]
Nejati 2021(1)	55.38	17.59	13	44.92	13.28	13	0.5%	0.65 [−0.14, 1.44]
Nejati 2022	45.67	14.82	24	47.43	16.56	24	1.0%	−0.11 [−0.68, 0.46]
Salehinejad 2020	21.979	23.106	17	27.825	26.457	17	0.7%	−0.23 [−0.90, 0.44]
**Subtotal (95% CI)**			**160**			**164**	**6.6%**	**−0.21 [−0.47, 0.05]**

Heterogeneity: $Tau^2 = 0.05$; $Chi^2 = 14.68$, df = 11 (P = 0.20); $I^2 = 25\%$

Test for overall effect: Z = 1.55 (P = 0.12)

3.1.6 drug

Allenby 2018	288.4	12.5	37	291.5	11.2	37	1.4%	−0.26 [−0.72, 0.20]
Cosmo 2015	0.83	17.14	30	2.13	20.41	30	1.2%	−0.07 [−0.57, 0.44]
Salehinejad 2022	16.21	19.51	22	19.875	27.132	22	0.9%	−0.15 [−0.74, 0.44]
Soltaninejad 2019	0.42	1.86	20	4.18	6.89	20	0.8%	−0.73 [−1.37, −0.09]
**Subtotal (95% CI)**			**109**			**109**	**4.3%**	**−0.27 [−0.53, 0.00]**

Heterogeneity: $Tau^2 = 0.00$; $Chi^2 = 2.74$, df = 3 (P = 0.43); $I^2 = 0\%$

Test for overall effect: Z = 1.94 (P = 0.05)

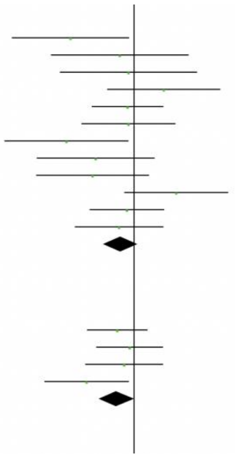

3.1.7 F3 or F4

Allenby 2018	288.4	12.5	37	291.5	11.2	37	1.4%	−0.26 [−0.72, 0.20]
Barham 2022	0.0001	0.0001	11	0.36	0.5	11	0.4%	−0.98 [−1.87, −0.08]
Cosmo 2015	0.83	17.14	30	2.13	20.41	30	1.2%	−0.07 [−0.57, 0.44]
D'Aiello 2023	20.81	15.16	26	22.64	20.68	26	1.1%	−0.10 [−0.64, 0.44]
Nejati 2020	80.14	11.42	15	81	7.8	15	0.6%	−0.09 [−0.80, 0.63]
Nejati 2020(2)	77.3	1.33	10	79.3	4.39	10	0.4%	−0.59 [−1.49, 0.31]
Nejati 2021	37.64	8.47	11	46.55	17	11	0.4%	−0.64 [−1.50, 0.22]
Nejati 2021(1)	55.38	17.59	13	44.92	13.28	13	0.5%	0.65 [−0.14, 1.44]
Nejati 2022	45.67	14.82	24	47.43	16.56	24	1.0%	−0.11 [−0.68, 0.46]
Salehinejad 2022	16.21	19.51	22	19.875	27.132	22	0.9%	−0.15 [−0.74, 0.44]
Soltaninejad 2019(1)	3.77	8.2	20	4.18	6.89	20	0.8%	−0.05 [−0.67, 0.57]
**Subtotal (95% CI)**			**219**			**219**	**8.9%**	**−0.17 [−0.36, 0.02]**

Heterogeneity: $Tau^2 = 0.00$; $Chi^2 = 9.83$, df = 10 (P = 0.46); $I^2 = 0\%$

Test for overall effect: Z = 1.74 (P = 0.08)

3.1.8 other targets

Breitling 2016	10.7	7.8	7	12.7	9.2	7	0.3%	−0.22 [−1.27, 0.83]
Breitling 2016(1)	11.9	8.3	7	12.7	9.2	7	0.3%	−0.09 [−1.13, 0.96]
Breitling–Ziegler 2021	26.02	18.13	9	18.83	12.73	13	0.4%	0.46 [−0.41, 1.32]
Nejati 2020 (1)	75.8	1.22	10	79.3	4.39	10	0.4%	−1.04 [−1.99, −0.09]
Soltaninejad 2019	0.42	1.86	20	4.18	6.89	20	0.8%	−0.73 [−1.37, −0.09]
**Subtotal (95% CI)**			**53**			**57**	**2.2%**	**−0.35 [−0.88, 0.18]**

Heterogeneity: $Tau^2 = 0.15$; $Chi^2 = 7.02$, df = 4 (P = 0.13); $I^2 = 43\%$

Test for overall effect: Z = 1.31 (P = 0.19)

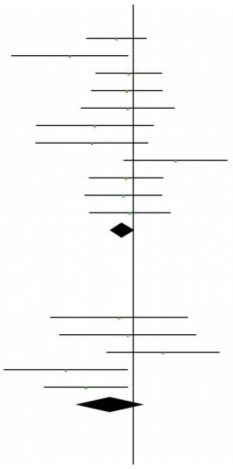

3.1.9 1 session

Breitling 2016	10.7	7.8	7	12.7	9.2	7	0.3%	−0.22 [−1.27, 0.83]
Breitling 2016(1)	11.9	8.3	7	12.7	9.2	7	0.3%	−0.09 [−1.13, 0.96]
Cosmo 2015	0.83	17.14	30	2.13	20.41	30	1.2%	−0.07 [−0.57, 0.44]
D'Aiello 2023	20.81	15.16	26	22.64	20.68	26	1.1%	−0.10 [−0.64, 0.44]
Nejati 2020	80.14	11.42	15	81	7.8	15	0.6%	−0.09 [−0.80, 0.63]
Nejati 2020 (1)	75.8	1.22	10	79.3	4.39	10	0.4%	−1.04 [−1.99, −0.09]
Nejati 2020(2)	77.3	1.33	10	79.3	4.39	10	0.4%	−0.59 [−1.49, 0.31]
Nejati 2021	37.64	8.47	11	46.55	17	11	0.4%	−0.64 [−1.50, 0.22]
Nejati 2021(1)	55.38	17.59	13	44.92	13.28	13	0.5%	0.65 [−0.14, 1.44]
Nejati 2022	45.67	14.82	24	47.43	16.56	24	1.0%	−0.11 [−0.68, 0.46]
Salehinejad 2020	21.979	23.106	17	27.825	26.457	17	0.7%	−0.23 [−0.90, 0.44]
Salehinejad 2022	16.21	19.51	22	19.875	27.132	22	0.9%	−0.15 [−0.74, 0.44]
Soltaninejad 2019	0.42	1.86	20	4.18	6.89	20	0.8%	−0.73 [−1.37, −0.09]
Soltaninejad 2019(1)	3.77	8.2	20	4.18	6.89	20	0.8%	−0.05 [−0.67, 0.57]
**Subtotal (95% CI)**			**232**			**232**	**9.5%**	**−0.20 [−0.39, −0.02]**

Heterogeneity: $Tau^2 = 0.00$; $Chi^2 = 12.66$, df = 13 (P = 0.47); $I^2 = 0\%$

Test for overall effect: Z = 2.15 (P = 0.03)

3.1.10 multi-sessions

Allenby 2018	288.4	12.5	37	291.5	11.2	37	1.4%	−0.26 [−0.72, 0.20]
Barham 2022	0.0001	0.0001	11	0.36	0.5	11	0.4%	−0.98 [−1.87, −0.08]
Breitling–Ziegler 2021	26.02	18.13	9	18.83	12.73	13	0.4%	0.46 [−0.41, 1.32]
**Subtotal (95% CI)**			**57**			**61**	**2.3%**	**−0.25 [−0.92, 0.42]**

Heterogeneity: $Tau^2 = 0.21$; $Chi^2 = 5.14$, df = 2 (P = 0.08); $I^2 = 61\%$

Test for overall effect: Z = 0.74 (P = 0.46)

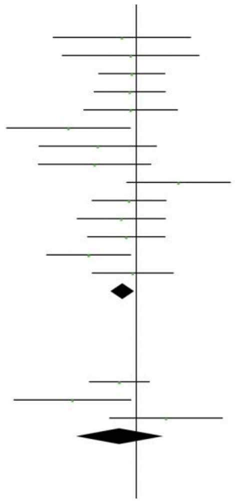

**3.1.11 offline**								
Barham 2022	0.0001	0.0001	11	0.36	0.5	11	0.4%	−0.98 [−1.87, −0.08]
Cosmo 2015	0.83	17.14	30	2.13	20.41	30	1.2%	−0.07 [−0.57, 0.44]
Nejati 2020	80.14	11.42	15	81	7.8	15	0.6%	−0.09 [−0.80, 0.63]
Nejati 2020 (1)	75.8	1.22	10	79.3	4.39	10	0.4%	−1.04 [−1.99, −0.09]
Nejati 2020(2)	77.3	1.33	10	79.3	4.39	10	0.4%	−0.59 [−1.49, 0.31]
Soltaninejad 2019	0.42	1.86	20	4.18	6.89	20	0.8%	−0.73 [−1.37, −0.09]
Soltaninejad 2019(1)	3.77	8.2	20	4.18	6.89	20	0.8%	−0.05 [−0.67, 0.57]
**Subtotal (95% CI)**			**116**			**116**	**4.6%**	**−0.41 [−0.72, −0.10]**

Heterogeneity: $Tau^2 = 0.05$; $Chi^2 = 8.12$, df = 6 (P = 0.23); $I^2 = 26\%$
Test for overall effect: Z = 2.57 (P = 0.01)

**3.1.12 online**								
Allenby 2018	288.4	12.5	37	291.5	11.2	37	1.4%	−0.26 [−0.72, 0.20]
Breitling 2016	10.7	7.8	7	12.7	9.2	7	0.3%	−0.22 [−1.27, 0.83]
Breitling 2016(1)	11.9	8.3	7	12.7	9.2	7	0.3%	−0.09 [−1.13, 0.96]
Breitling-Ziegler 2021	26.02	18.13	9	18.83	12.73	13	0.4%	0.46 [−0.41, 1.32]
D'Aiello 2023	20.81	15.16	26	22.64	20.68	26	1.1%	−0.10 [−0.64, 0.44]
Nejati 2021	0	0	0	0	0	0		Not estimable
Nejati 2021(1)	0	0	0	0	0	0		Not estimable
Nejati 2022	0	0	0	0	0	0		Not estimable
Salehinejad 2020	0	0	0	0	0	0		Not estimable
Salehinejad 2022	16.21	19.51	22	19.875	27.132	22	0.9%	−0.15 [−0.74, 0.44]
**Subtotal (95% CI)**			**108**			**112**	**4.5%**	**−0.12 [−0.38, 0.15]**

Heterogeneity: $Tau^2 = 0.00$; $Chi^2 = 2.13$, df = 5 (P = 0.83); $I^2 = 0\%$
Test for overall effect: Z = 0.87 (P = 0.39)

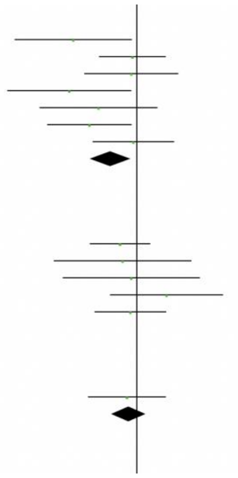

**3.1.13 15 min**								
Nejati 2020	80.14	11.42	15	81	7.8	15	0.6%	−0.09 [−0.80, 0.63]
Nejati 2020 (1)	75.8	1.22	10	79.3	4.39	10	0.4%	−1.04 [−1.99, −0.09]
Nejati 2020(2)	77.3	1.33	10	79.3	4.39	10	0.4%	−0.59 [−1.49, 0.31]
Salehinejad 2022	16.21	19.51	22	19.875	27.132	22	0.9%	−0.15 [−0.74, 0.44]
Soltaninejad 2019	0.42	1.86	20	4.18	6.89	20	0.8%	−0.73 [−1.37, −0.09]
Soltaninejad 2019(1)	3.77	8.2	20	4.18	6.89	20	0.8%	−0.05 [−0.67, 0.57]
**Subtotal (95% CI)**			**97**			**97**	**3.9%**	**−0.37 [−0.67, −0.06]**

Heterogeneity: $Tau^2 = 0.01$; $Chi^2 = 5.49$, df = 5 (P = 0.36); $I^2 = 9\%$
Test for overall effect: Z = 2.38 (P = 0.02)

**3.1.14 20 min**								
Allenby 2018	288.4	12.5	37	291.5	11.2	37	1.4%	−0.26 [−0.72, 0.20]
Barham 2022	0.0001	0.0001	11	0.36	0.5	11	0.4%	−0.98 [−1.87, −0.08]
Breitling 2016	10.7	7.8	7	12.7	9.2	7	0.3%	−0.22 [−1.27, 0.83]
Breitling 2016(1)	11.9	8.3	7	12.7	9.2	7	0.3%	−0.09 [−1.13, 0.96]
Breitling-Ziegler 2021	26.02	18.13	9	18.83	12.73	13	0.4%	0.46 [−0.41, 1.32]
Cosmo 2015	0.83	17.14	30	2.13	20.41	30	1.2%	−0.07 [−0.57, 0.44]
D'Aiello 2023	20.81	15.16	26	22.64	20.68	26	1.1%	−0.10 [−0.64, 0.44]
Nejati 2021	37.64	8.47	11	46.55	17	11	0.4%	−0.64 [−1.50, 0.22]
Nejati 2021(1)	55.38	17.59	13	44.92	13.28	13	0.5%	0.65 [−0.14, 1.44]
Nejati 2022	45.67	14.82	24	47.43	16.56	24	1.0%	−0.11 [−0.68, 0.46]
Salehinejad 2020	21.979	23.106	17	27.825	26.457	17	0.7%	−0.23 [−0.90, 0.44]
**Subtotal (95% CI)**			**192**			**196**	**7.8%**	**−0.14 [−0.35, 0.07]**

Heterogeneity: $Tau^2 = 0.01$; $Chi^2 = 10.79$, df = 10 (P = 0.37); $I^2 = 7\%$
Test for overall effect: Z = 1.27 (P = 0.21)

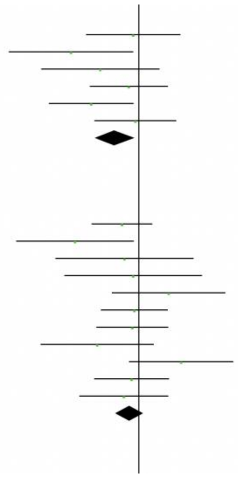

**3.1.15 Crossover**								
Allenby 2018	288.4	12.5	37	291.5	11.2	37	1.4%	−0.26 [−0.72, 0.20]
D'Aiello 2023	20.81	15.16	26	22.64	20.68	26	1.1%	−0.10 [−0.64, 0.44]
Nejati 2020	80.14	11.42	15	81	7.8	15	0.6%	−0.09 [−0.80, 0.63]
Nejati 2020 (1)	75.8	1.22	10	79.3	4.39	10	0.4%	−1.04 [−1.99, −0.09]
Nejati 2020(2)	77.3	1.33	10	79.3	4.39	10	0.4%	−0.59 [−1.49, 0.31]
Nejati 2021	37.64	8.47	11	46.55	17	11	0.4%	−0.64 [−1.50, 0.22]
Nejati 2021(1)	55.38	17.59	13	44.92	13.28	13	0.5%	0.65 [−0.14, 1.44]
Nejati 2022	45.67	14.82	24	47.43	16.56	24	1.0%	−0.11 [−0.68, 0.46]
Salehinejad 2020	21.979	23.106	17	27.825	26.457	17	0.7%	−0.23 [−0.90, 0.44]
Salehinejad 2022	16.21	19.51	22	19.875	27.132	22	0.9%	−0.15 [−0.74, 0.44]
Soltaninejad 2019	0.42	1.86	20	4.18	6.89	20	0.8%	−0.73 [−1.37, −0.09]
Soltaninejad 2019(1)	3.77	8.2	20	4.18	6.89	20	0.8%	−0.05 [−0.67, 0.57]
**Subtotal (95% CI)**			**225**			**225**	**9.1%**	**−0.23 [−0.43, −0.04]**

Heterogeneity: $Tau^2 = 0.01$; $Chi^2 = 12.30$, df = 11 (P = 0.34); $I^2 = 11\%$
Test for overall effect: Z = 2.31 (P = 0.02)

**3.1.16 Parallel**								
Barham 2022	0.0001	0.0001	11	0.36	0.5	11	0.4%	−0.98 [−1.87, −0.08]
Breitling 2016	10.7	7.8	7	12.7	9.2	7	0.3%	−0.22 [−1.27, 0.83]
Breitling 2016(1)	11.9	8.3	7	12.7	9.2	7	0.3%	−0.09 [−1.13, 0.96]
Breitling-Ziegler 2021	26.02	18.13	9	18.83	12.73	13	0.4%	0.46 [−0.41, 1.32]
Cosmo 2015	0.83	17.14	30	2.13	20.41	30	1.2%	−0.07 [−0.57, 0.44]
**Subtotal (95% CI)**			**64**			**68**	**2.7%**	**−0.15 [−0.58, 0.27]**

Heterogeneity: $Tau^2 = 0.06$; $Chi^2 = 5.33$, df = 4 (P = 0.25); $I^2 = 25\%$
Test for overall effect: Z = 0.71 (P = 0.48)

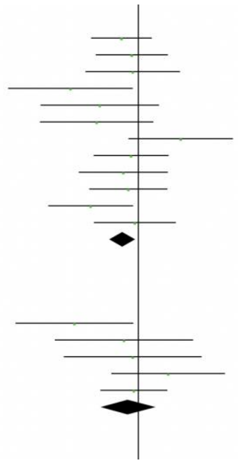

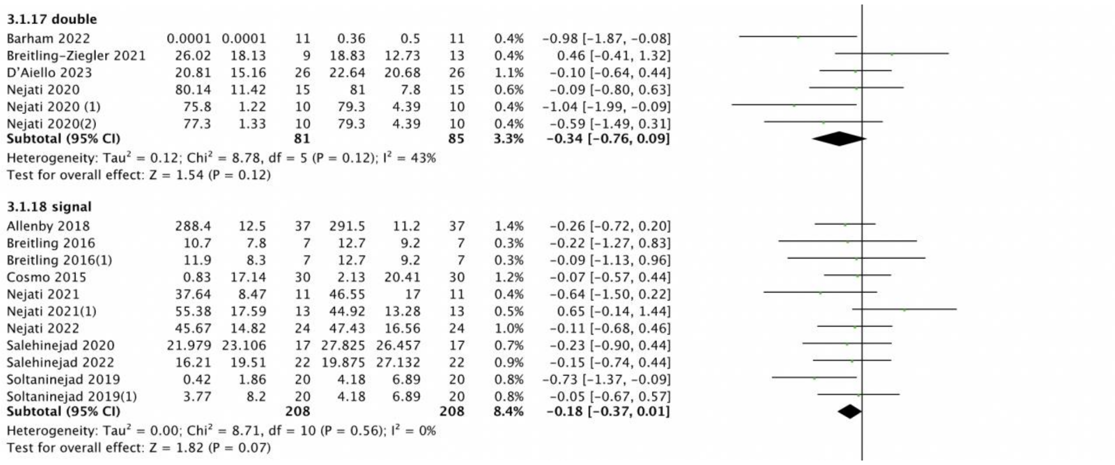

Figure S1 Subgroup analysis of inhibition in tDCS studies. In subgroup analyses for offline and online interventions, studies categorized as 'not estimable' represent interventions that combine elements from both offline and online approaches, not strictly belonging to either category.

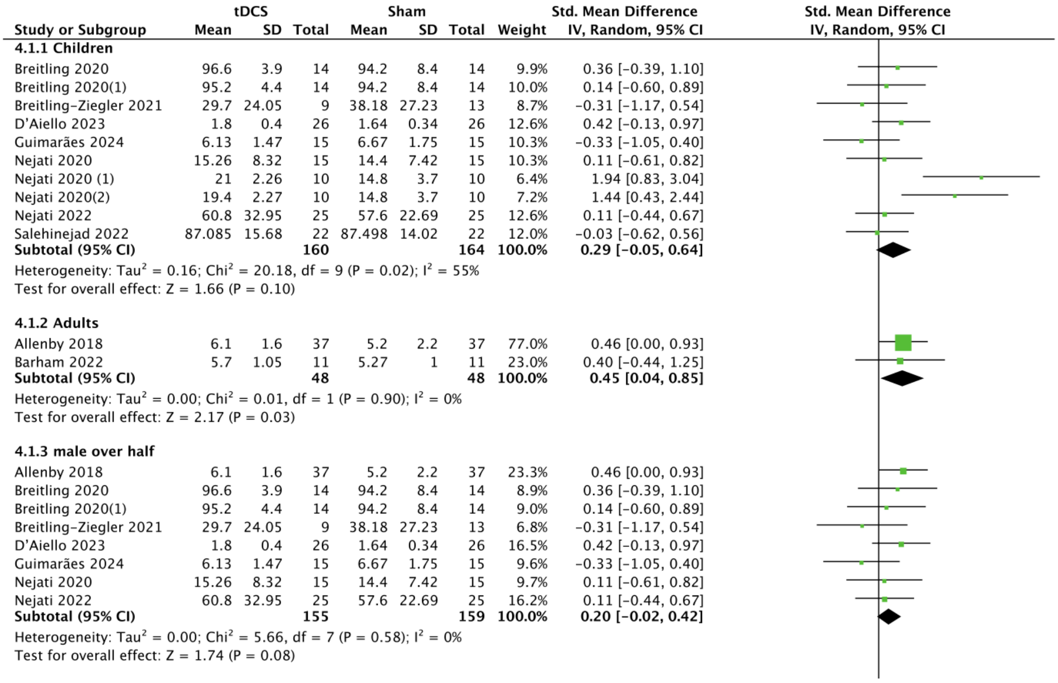

**4.1.4 male less half**								
Barham 2022	5.7	1.05	11	5.27	1	11	25.6%	0.40 [−0.44, 1.25]
Nejati 2020 (1)	21	2.26	10	14.8	3.7	10	22.0%	1.94 [0.83, 3.04]
Nejati 2020(2)	19.4	2.27	10	14.8	3.7	10	23.3%	1.44 [0.43, 2.44]
Salehinejad 2022	87.085	15.68	22	87.498	14.02	22	29.0%	−0.03 [−0.62, 0.56]
**Subtotal (95% CI)**			**53**			**53**	**100.0%**	**0.86 [−0.03, 1.75]**

Heterogeneity: $Tau^2 = 0.62$; $Chi^2 = 12.80$, df = 3 (P = 0.005); $I^2 = 77\%$
Test for overall effect: Z = 1.89 (P = 0.06)

**4.1.5 drug−naive**								
Barham 2022	5.7	1.05	11	5.27	1	11	9.1%	0.40 [−0.44, 1.25]
Breitling 2020	96.6	3.9	14	94.2	8.4	14	10.3%	0.36 [−0.39, 1.10]
Breitling 2020(1)	95.2	4.4	14	94.2	8.4	14	10.3%	0.14 [−0.60, 0.89]
Breitling−Ziegler 2021	29.7	24.05	9	38.18	27.23	13	9.0%	−0.31 [−1.17, 0.54]
D'Aiello 2023	1.8	0.4	26	1.64	0.34	26	13.1%	0.42 [−0.13, 0.97]
Guimarães 2024	6.13	1.47	15	6.67	1.75	15	10.6%	−0.33 [−1.05, 0.40]
Nejati 2020	15.26	8.32	15	14.4	7.42	15	10.7%	0.11 [−0.61, 0.82]
Nejati 2020 (1)	21	2.26	10	14.8	3.7	10	6.6%	1.94 [0.83, 3.04]
Nejati 2020(2)	19.4	2.27	10	14.8	3.7	10	7.4%	1.44 [0.43, 2.44]
Nejati 2022	60.8	32.95	25	57.6	22.69	25	13.0%	0.11 [−0.44, 0.67]
**Subtotal (95% CI)**			**149**			**153**	**100.0%**	**0.34 [−0.01, 0.69]**

Heterogeneity: $Tau^2 = 0.16$; $Chi^2 = 19.36$, df = 9 (P = 0.02); $I^2 = 54\%$
Test for overall effect: Z = 1.91 (P = 0.06)

**4.1.7 F3 or F4**								
Allenby 2018	6.1	1.6	37	5.2	2.2	37	17.1%	0.46 [0.00, 0.93]
Barham 2022	5.7	1.05	11	5.27	1	11	9.5%	0.40 [−0.44, 1.25]
D'Aiello 2023	1.8	0.4	26	1.64	0.34	26	15.0%	0.42 [−0.13, 0.97]
Guimarães 2024	6.13	1.47	15	6.67	1.75	15	11.5%	−0.33 [−1.05, 0.40]
Nejati 2020	15.26	8.32	15	14.4	7.42	15	11.5%	0.11 [−0.61, 0.82]
Nejati 2020 (1)	21	2.26	10	14.8	3.7	10	6.5%	1.94 [0.83, 3.04]
Nejati 2022	60.8	32.95	25	57.6	22.69	25	14.9%	0.11 [−0.44, 0.67]
Salehinejad 2022	87.085	15.68	22	87.498	14.02	22	14.0%	−0.03 [−0.62, 0.56]
**Subtotal (95% CI)**			**161**			**161**	**100.0%**	**0.30 [−0.03, 0.62]**

Heterogeneity: $Tau^2 = 0.11$; $Chi^2 = 13.94$, df = 7 (P = 0.05); $I^2 = 50\%$
Test for overall effect: Z = 1.78 (P = 0.08)

**4.1.8 F8 or Fp2**								
Breitling 2020	96.6	3.9	14	94.2	8.4	14	27.3%	0.36 [−0.39, 1.10]
Breitling 2020(1)	95.2	4.4	14	94.2	8.4	14	27.4%	0.14 [−0.60, 0.89]
Breitling−Ziegler 2021	29.7	24.05	9	38.18	27.23	13	24.4%	−0.31 [−1.17, 0.54]
Nejati 2020(2)	19.4	2.27	10	14.8	3.7	10	20.9%	1.44 [0.43, 2.44]
**Subtotal (95% CI)**			**47**			**51**	**100.0%**	**0.36 [−0.28, 0.99]**

Heterogeneity: $Tau^2 = 0.24$; $Chi^2 = 7.04$, df = 3 (P = 0.07); $I^2 = 57\%$
Test for overall effect: Z = 1.11 (P = 0.27)

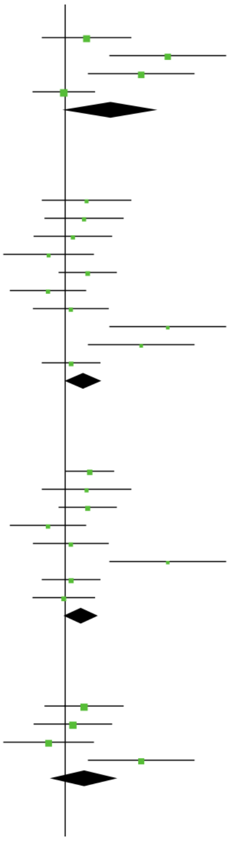

**4.1.9 1 sessions**								
Breitling 2020	96.6	3.9	14	94.2	8.4	14	12.2%	0.36 [−0.39, 1.10]
Breitling 2020(1)	95.2	4.4	14	94.2	8.4	14	12.3%	0.14 [−0.60, 0.89]
D'Aiello 2023	1.8	0.4	26	1.64	0.34	26	15.7%	0.42 [−0.13, 0.97]
Nejati 2020	15.26	8.32	15	14.4	7.42	15	12.7%	0.11 [−0.61, 0.82]
Nejati 2020 (1)	21	2.26	10	14.8	3.7	10	7.8%	1.94 [0.83, 3.04]
Nejati 2020(2)	19.4	2.27	10	14.8	3.7	10	8.8%	1.44 [0.43, 2.44]
Nejati 2022	60.8	32.95	25	57.6	22.69	25	15.6%	0.11 [−0.44, 0.67]
Salehinejad 2022	87.085	15.68	22	87.498	14.02	22	14.9%	−0.03 [−0.62, 0.56]
**Subtotal (95% CI)**			**136**			**136**	**100.0%**	**0.43 [0.06, 0.81]**

Heterogeneity: $Tau^2 = 0.15$; $Chi^2 = 15.49$, df = 7 (P = 0.03); $I^2 = 55\%$
Test for overall effect: Z = 2.25 (P = 0.02)

**4.1.10 multi−sessions**								
Allenby 2018	6.1	1.6	37	5.2	2.2	37	38.0%	0.46 [0.00, 0.93]
Barham 2022	5.7	1.05	11	5.27	1	11	19.2%	0.40 [−0.44, 1.25]
Breitling−Ziegler 2021	29.7	24.05	9	38.18	27.23	13	18.9%	−0.31 [−1.17, 0.54]
Guimarães 2024	6.13	1.47	15	6.67	1.75	15	23.8%	−0.33 [−1.05, 0.40]
**Subtotal (95% CI)**			**72**			**76**	**100.0%**	**0.12 [−0.33, 0.56]**

Heterogeneity: $Tau^2 = 0.08$; $Chi^2 = 4.87$, df = 3 (P = 0.18); $I^2 = 38\%$
Test for overall effect: Z = 0.52 (P = 0.60)

**4.1.11 offline**								
Barham 2022	5.7	1.05	11	5.27	1	11	17.0%	0.40 [−0.44, 1.25]
Breitling−Ziegler 2021	29.7	24.05	9	38.18	27.23	13	16.9%	−0.31 [−1.17, 0.54]
Guimarães 2024	6.13	1.47	15	6.67	1.75	15	18.3%	−0.33 [−1.05, 0.40]
Nejati 2020	15.26	8.32	15	14.4	7.42	15	18.4%	0.11 [−0.61, 0.82]
Nejati 2020 (1)	21	2.26	10	14.8	3.7	10	14.3%	1.94 [0.83, 3.04]
Nejati 2020(2)	19.4	2.27	10	14.8	3.7	10	15.3%	1.44 [0.43, 2.44]
**Subtotal (95% CI)**			**70**			**74**	**100.0%**	**0.47 [−0.20, 1.14]**

Heterogeneity: $Tau^2 = 0.51$; $Chi^2 = 18.52$, df = 5 (P = 0.002); $I^2 = 73\%$
Test for overall effect: Z = 1.37 (P = 0.17)

**4.1.12 online**								
Allenby 2018	6.1	1.6	37	5.2	2.2	37	26.5%	0.46 [0.00, 0.93]
Breitling 2020	96.6	3.9	14	94.2	8.4	14	10.1%	0.36 [−0.39, 1.10]
Breitling 2020(1)	96.6	3.9	14	94.2	8.4	14	10.1%	0.36 [−0.39, 1.10]
D'Aiello 2023	1.8	0.4	26	1.64	0.34	26	18.7%	0.42 [−0.13, 0.97]
Nejati 2022	60.8	32.95	25	57.6	22.69	25	18.4%	0.11 [−0.44, 0.67]
Salehinejad 2022	87.085	15.68	22	87.498	14.02	22	16.2%	−0.03 [−0.62, 0.56]
**Subtotal (95% CI)**			**138**			**138**	**100.0%**	**0.29 [0.05, 0.53]**

Heterogeneity: $Tau^2 = 0.00$; $Chi^2 = 2.33$, df = 5 (P = 0.80); $I^2 = 0\%$
Test for overall effect: Z = 2.39 (P = 0.02)

**4.1.13 15 min**								
Nejati 2020	15.26	8.32	15	14.4	7.42	15	26.9%	0.11 [−0.61, 0.82]
Nejati 2020 (1)	21	2.26	10	14.8	3.7	10	21.7%	1.94 [0.83, 3.04]
Nejati 2020(2)	19.4	2.27	10	14.8	3.7	10	23.0%	1.44 [0.43, 2.44]
Salehinejad 2022	87.085	15.68	22	87.498	14.02	22	28.5%	−0.03 [−0.62, 0.56]
**Subtotal (95% CI)**			**57**			**57**	**100.0%**	**0.77 [−0.12, 1.66]**

Heterogeneity: $Tau^2 = 0.63$; $Chi^2 = 14.03$, df = 3 (P = 0.003); $I^2 = 79\%$
Test for overall effect: Z = 1.70 (P = 0.09)

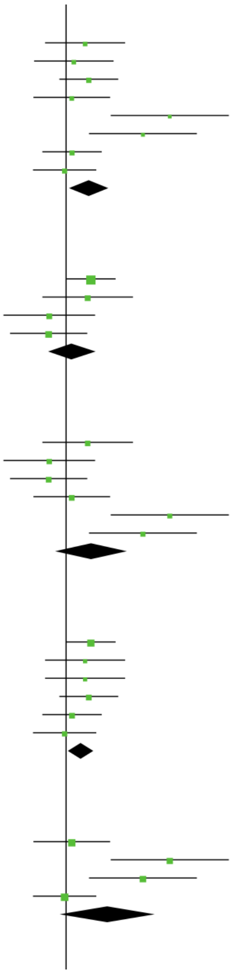

**4.1.14 20 min**								
Allenby 2018	6.1	1.6	37	5.2	2.2	37	24.0%	0.46 [0.00, 0.93]
Barham 2022	5.7	1.05	11	5.27	1	11	7.2%	0.40 [−0.44, 1.25]
Breitling 2020	96.6	3.9	14	94.2	8.4	14	9.2%	0.36 [−0.39, 1.10]
Breitling 2020(1)	95.2	4.4	14	94.2	8.4	14	9.3%	0.14 [−0.60, 0.89]
Breitling–Ziegler 2021	29.7	24.05	9	38.18	27.23	13	7.0%	−0.31 [−1.17, 0.54]
D'Aiello 2023	1.8	0.4	26	1.64	0.34	26	16.9%	0.42 [−0.13, 0.97]
Guimarães 2024	6.13	1.47	15	6.67	1.75	15	9.8%	−0.33 [−1.05, 0.40]
Nejati 2022	60.8	32.95	25	57.6	22.69	25	16.6%	0.11 [−0.44, 0.67]
**Subtotal (95% CI)**			**151**			**155**	**100.0%**	**0.22 [−0.00, 0.45]**
Heterogeneity: $Tau^2 = 0.00$; $Chi^2 = 5.78$, df = 7 (P = 0.57); $I^2 = 0\%$								
Test for overall effect: Z = 1.93 (P = 0.05)								
**4.1.15 crossover**								
Allenby 2018	6.1	1.6	37	5.2	2.2	37	13.8%	0.46 [0.00, 0.93]
Breitling 2020	96.6	3.9	14	94.2	8.4	14	9.3%	0.36 [−0.39, 1.10]
Breitling 2020(1)	95.2	4.4	14	94.2	8.4	14	9.3%	0.14 [−0.60, 0.89]
D'Aiello 2023	1.8	0.4	26	1.64	0.34	26	12.3%	0.42 [−0.13, 0.97]
Guimarães 2024	6.13	1.47	15	6.67	1.75	15	9.6%	−0.33 [−1.05, 0.40]
Nejati 2020	21	2.26	10	14.8	3.7	10	5.7%	1.94 [0.83, 3.04]
Nejati 2020 (1)	15.26	8.32	15	14.4	7.42	15	9.7%	0.11 [−0.61, 0.82]
Nejati 2020(2)	19.4	2.27	10	14.8	3.7	10	6.5%	1.44 [0.43, 2.44]
Nejati 2022	60.8	32.95	25	57.6	22.69	25	12.2%	0.11 [−0.44, 0.67]
Salehinejad 2022	87.085	15.68	22	87.498	14.02	22	11.6%	−0.03 [−0.62, 0.56]
**Subtotal (95% CI)**			**188**			**188**	**100.0%**	**0.35 [0.04, 0.67]**
Heterogeneity: $Tau^2 = 0.13$; $Chi^2 = 18.97$, df = 9 (P = 0.03); $I^2 = 53\%$								
Test for overall effect: Z = 2.24 (P = 0.03)								

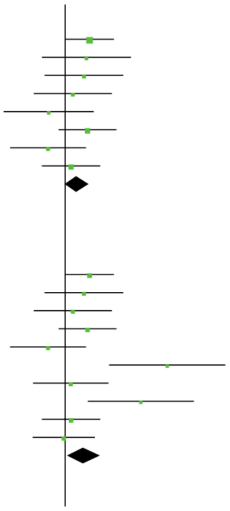

Figure S2 Subgroup analysis of working memory in tDCS studies. In subgroup analyses for offline and online interventions, studies categorized as 'not estimable' represent interventions that combine elements from both offline and online approaches, not strictly belonging to either category. Since there are 9 studies involving drug-naive participants and 2 studies with unspecified drug conditions among those investigating tDCS and working memory, subgroup analysis of the medication group is not conducted here.

Study or Subgroup	tDCS Mean	tDCS SD	tDCS Total	Sham Mean	Sham SD	Sham Total	Weight	Std. Mean Difference IV, Random, 95% CI	Year
**3.1.1 Children**									
Soff 2017	1.79	0.46	15	1.46	0.57	15	49.3%	0.62 [−0.12, 1.36]	2016
Guimarães 2024	7.87	24.3	15	10	27.75	15	50.7%	−0.08 [−0.80, 0.64]	2024
**Subtotal (95% CI)**			**30**			**30**	**100.0%**	**0.27 [−0.42, 0.95]**	
Heterogeneity: $Tau^2 = 0.11$; $Chi^2 = 1.78$, df = 1 (P = 0.18); $I^2 = 44\%$									
Test for overall effect: Z = 0.76 (P = 0.45)									
**3.1.2 Alduts**									
Cachoeira 2017	20.78	2.19	9	26.25	1.92	8	13.1%	−2.51 [−3.86, −1.16]	2016
Allenby 2018	17.1	1.5	37	19.8	1.8	37	23.0%	−1.61 [−2.14, −1.08]	2018
Jacoby 2018	541.51	50.83	20	546.96	53.48	20	21.9%	−0.10 [−0.72, 0.52]	2018
Leffa 2022	18.88	5.79	32	23.63	3.97	32	23.1%	−0.95 [−1.46, −0.43]	2022
Barham 2022	0.81	1.07	11	1.4	1.34	11	18.9%	−0.47 [−1.32, 0.38]	2022
**Subtotal (95% CI)**			**109**			**108**	**100.0%**	**−1.03 [−1.71, −0.34]**	
Heterogeneity: $Tau^2 = 0.46$; $Chi^2 = 19.52$, df = 4 (P = 0.0006); $I^2 = 80\%$									
Test for overall effect: Z = 2.94 (P = 0.003)									
**3.1.3 male more half**									
Soff 2017	1.79	0.46	15	1.46	0.57	15	24.0%	0.62 [−0.12, 1.36]	2016
Allenby 2018	17.1	1.5	37	19.8	1.8	37	25.9%	−1.61 [−2.14, −1.08]	2018
Leffa 2022	18.88	5.79	32	23.63	3.97	32	26.0%	−0.95 [−1.46, −0.43]	2022
Guimarães 2024	7.87	24.3	15	10	27.75	15	24.2%	−0.08 [−0.80, 0.64]	2024
**Subtotal (95% CI)**			**99**			**99**	**100.0%**	**−0.53 [−1.46, 0.39]**	
Heterogeneity: $Tau^2 = 0.79$; $Chi^2 = 27.50$, df = 3 (P < 0.00001); $I^2 = 89\%$									
Test for overall effect: Z = 1.13 (P = 0.26)									
**3.1.4 male less half**									
Cachoeira 2017	20.78	2.19	9	26.25	1.92	8	27.1%	−2.51 [−3.86, −1.16]	2016
Jacoby 2018	541.51	50.83	20	546.96	53.48	20	38.1%	−0.10 [−0.72, 0.52]	2018
Barham 2022	0.81	1.07	11	1.4	1.34	11	34.8%	−0.47 [−1.32, 0.38]	2022
**Subtotal (95% CI)**			**40**			**39**	**100.0%**	**−0.88 [−2.04, 0.28]**	
Heterogeneity: $Tau^2 = 0.82$; $Chi^2 = 10.05$, df = 2 (P = 0.007); $I^2 = 80\%$									
Test for overall effect: Z = 1.49 (P = 0.14)									
**3.1.5 drug naive**									
Soff 2017	1.79	0.46	15	1.46	0.57	15	24.3%	0.62 [−0.12, 1.36]	2016
Jacoby 2018	541.51	50.83	20	546.96	53.48	20	31.0%	−0.10 [−0.72, 0.52]	2018
Barham 2022	0.81	1.07	11	1.4	1.34	11	19.4%	−0.47 [−1.32, 0.38]	2022
Guimarães 2024	7.87	24.3	15	10	27.75	15	25.3%	−0.08 [−0.80, 0.64]	2024
**Subtotal (95% CI)**			**61**			**61**	**100.0%**	**0.01 [−0.41, 0.43]**	
Heterogeneity: $Tau^2 = 0.05$; $Chi^2 = 4.05$, df = 3 (P = 0.26); $I^2 = 26\%$									
Test for overall effect: Z = 0.04 (P = 0.97)									
**3.1.6 drug**									
Cachoeira 2017	20.78	2.19	9	26.25	1.92	8	41.7%	−2.51 [−3.86, −1.16]	2016
Leffa 2022	18.88	5.79	32	23.63	3.97	32	58.3%	−0.95 [−1.46, −0.43]	2022
**Subtotal (95% CI)**			**41**			**40**	**100.0%**	**−1.60 [−3.11, −0.09]**	
Heterogeneity: $Tau^2 = 0.95$; $Chi^2 = 4.47$, df = 1 (P = 0.03); $I^2 = 78\%$									
Test for overall effect: Z = 2.07 (P = 0.04)									

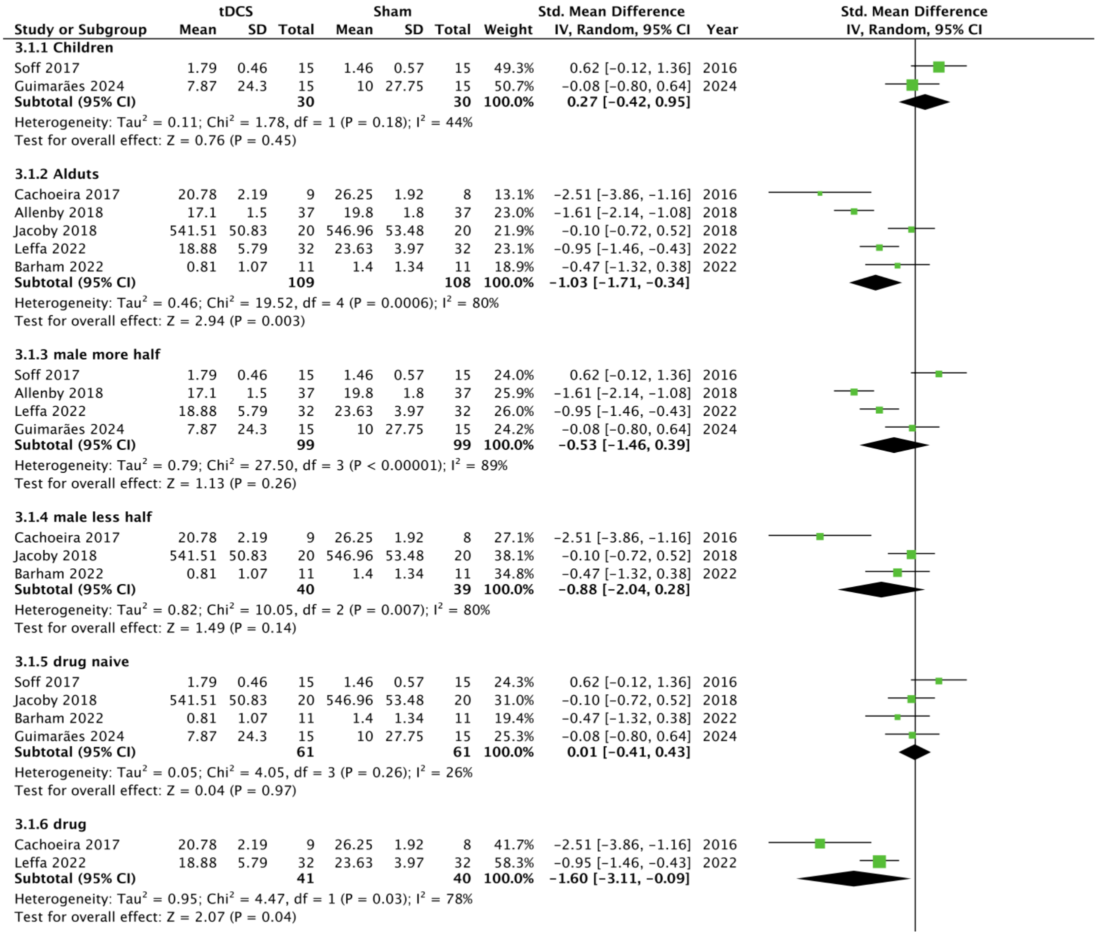

**3.1.7 F3 and F4**									
Cachoeira 2017	20.78	2.19	9	26.25	1.92	8	10.2%	−2.51 [−3.86, −1.16]	2016
Soff 2017	1.79	0.46	15	1.46	0.57	15	14.5%	0.62 [−0.12, 1.36]	2016
Jacoby 2018	541.51	50.83	20	546.96	53.48	20	15.3%	−0.10 [−0.72, 0.52]	2018
Allenby 2018	17.1	1.5	37	19.8	1.8	37	15.9%	−1.61 [−2.14, −1.08]	2018
Barham 2022	0.81	1.07	11	1.4	1.34	11	13.7%	−0.47 [−1.32, 0.38]	2022
Leffa 2022	18.88	5.79	32	23.63	3.97	32	15.9%	−0.95 [−1.46, −0.43]	2022
Guimarães 2024	7.87	24.3	15	10	27.75	15	14.6%	−0.08 [−0.80, 0.64]	2024
**Subtotal (95% CI)**			**139**			**138**	**100.0%**	**−0.66 [−1.33, 0.00]**	
Heterogeneity: Tau² = 0.65; Chi² = 38.28, df = 6 (P < 0.00001); I² = 84%									
Test for overall effect: Z = 1.96 (P = 0.05)									
**3.1.8 other targets**									
**Subtotal (95% CI)**			**0**			**0**		**Not estimable**	
Heterogeneity: Not applicable									
Test for overall effect: Not applicable									
**3.1.9 1 session**									
Jacoby 2018	541.51	50.83	20	546.96	53.48	20	100.0%	−0.10 [−0.72, 0.52]	2018
**Subtotal (95% CI)**			**20**			**20**	**100.0%**	**−0.10 [−0.72, 0.52]**	
Heterogeneity: Not applicable									
Test for overall effect: Z = 0.32 (P = 0.75)									
**3.1.10 multi-sessions**									
Cachoeira 2017	20.78	2.19	9	26.25	1.92	8	12.4%	−2.51 [−3.86, −1.16]	2016
Soff 2017	1.79	0.46	15	1.46	0.57	15	17.1%	0.62 [−0.12, 1.36]	2016
Allenby 2018	17.1	1.5	37	19.8	1.8	37	18.5%	−1.61 [−2.14, −1.08]	2018
Leffa 2022	18.88	5.79	32	23.63	3.97	32	18.6%	−0.95 [−1.46, −0.43]	2022
Barham 2022	0.81	1.07	11	1.4	1.34	11	16.2%	−0.47 [−1.32, 0.38]	2022
Guimarães 2024	7.87	24.3	15	10	27.75	15	17.2%	−0.08 [−0.80, 0.64]	2024
**Subtotal (95% CI)**			**119**			**118**	**100.0%**	**−0.77 [−1.53, −0.01]**	
Heterogeneity: Tau² = 0.74; Chi² = 34.32, df = 5 (P < 0.00001); I² = 85%									
Test for overall effect: Z = 1.98 (P = 0.05)									
**3.1.11 offline**									
Cachoeira 2017	20.78	2.19	9	26.25	1.92	8	12.3%	−2.51 [−3.86, −1.16]	2016
Jacoby 2018	541.51	50.83	20	546.96	53.48	20	22.9%	−0.10 [−0.72, 0.52]	2018
Barham 2022	0.81	1.07	11	1.4	1.34	11	19.1%	−0.47 [−1.32, 0.38]	2022
Leffa 2022	18.88	5.79	32	23.63	3.97	32	24.5%	−0.95 [−1.46, −0.43]	2022
Guimarães 2024	7.87	24.3	15	10	27.75	15	21.2%	−0.08 [−0.80, 0.64]	2024
**Subtotal (95% CI)**			**87**			**86**	**100.0%**	**−0.67 [−1.29, −0.05]**	
Heterogeneity: Tau² = 0.34; Chi² = 13.93, df = 4 (P = 0.008); I² = 71%									
Test for overall effect: Z = 2.11 (P = 0.03)									
**3.1.12 online**									
Soff 2017	1.79	0.46	15	1.46	0.57	15	49.3%	0.62 [−0.12, 1.36]	2016
Allenby 2018	17.1	1.5	37	19.8	1.8	37	50.7%	−1.61 [−2.14, −1.08]	2018
**Subtotal (95% CI)**			**52**			**52**	**100.0%**	**−0.51 [−2.70, 1.68]**	
Heterogeneity: Tau² = 2.39; Chi² = 23.37, df = 1 (P < 0.00001); I² = 96%									
Test for overall effect: Z = 0.46 (P = 0.65)									

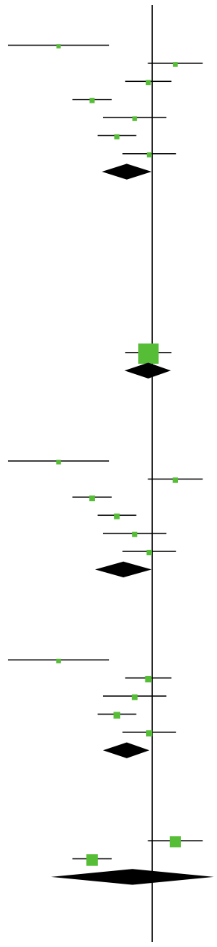

**3.1.13 crossover**									
Soff 2017	1.79	0.46	15	1.46	0.57	15	24.3%	0.62 [−0.12, 1.36]	2016
Jacoby 2018	541.51	50.83	20	546.96	53.48	20	25.3%	−0.10 [−0.72, 0.52]	2018
Allenby 2018	17.1	1.5	37	19.8	1.8	37	26.0%	−1.61 [−2.14, −1.08]	2018
Guimarães 2024	7.87	24.3	15	10	27.75	15	24.5%	−0.08 [−0.80, 0.64]	2024
**Subtotal (95% CI)**			**87**			**87**	**100.0%**	**−0.31 [−1.31, 0.68]**	
Heterogeneity: Tau² = 0.92; Chi² = 28.86, df = 3 (P < 0.00001); I² = 90%									
Test for overall effect: Z = 0.62 (P = 0.54)									
**3.1.14 parallel**									
Cachoeira 2017	20.78	2.19	9	26.25	1.92	8	22.9%	−2.51 [−3.86, −1.16]	2016
Barham 2022	0.81	1.07	11	1.4	1.34	11	34.2%	−0.47 [−1.32, 0.38]	2022
Leffa 2022	18.88	5.79	32	23.63	3.97	32	42.8%	−0.95 [−1.46, −0.43]	2022
**Subtotal (95% CI)**			**52**			**51**	**100.0%**	**−1.14 [−2.02, −0.26]**	
Heterogeneity: Tau² = 0.40; Chi² = 6.32, df = 2 (P = 0.04); I² = 68%									
Test for overall effect: Z = 2.54 (P = 0.01)									
**3.1.15 double blind**									
Soff 2017	1.79	0.46	15	1.46	0.57	15	24.5%	0.62 [−0.12, 1.36]	2016
Leffa 2022	18.88	5.79	32	23.63	3.97	32	28.3%	−0.95 [−1.46, −0.43]	2022
Barham 2022	0.81	1.07	11	1.4	1.34	11	22.5%	−0.47 [−1.32, 0.38]	2022
Guimarães 2024	7.87	24.3	15	10	27.75	15	24.8%	−0.08 [−0.80, 0.64]	2024
**Subtotal (95% CI)**			**73**			**73**	**100.0%**	**−0.24 [−0.94, 0.46]**	
Heterogeneity: Tau² = 0.39; Chi² = 12.37, df = 3 (P = 0.006); I² = 76%									
Test for overall effect: Z = 0.67 (P = 0.50)									
**3.1.16 single blind**									
Cachoeira 2017	20.78	2.19	9	26.25	1.92	8	27.3%	−2.51 [−3.86, −1.16]	2016
Jacoby 2018	541.51	50.83	20	546.96	53.48	20	35.9%	−0.10 [−0.72, 0.52]	2018
Allenby 2018	17.1	1.5	37	19.8	1.8	37	36.8%	−1.61 [−2.14, −1.08]	2018
**Subtotal (95% CI)**			**66**			**65**	**100.0%**	**−1.31 [−2.59, −0.04]**	
Heterogeneity: Tau² = 1.09; Chi² = 17.72, df = 2 (P = 0.0001); I² = 89%									
Test for overall effect: Z = 2.01 (P = 0.04)									

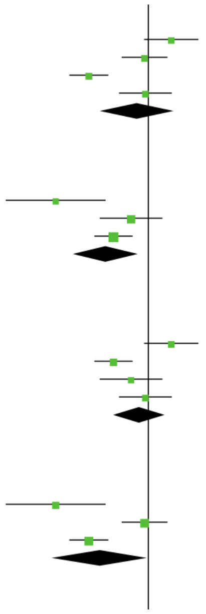

Figure S3 Subgroup analysis of inattention in tDCS studies. In subgroup analyses for offline and online interventions, studies categorized as 'not estimable' represent interventions that combine elements from both offline and online approaches, not strictly belonging to either category. Since all studies related to attention have treatment duration of 20 minutes each

session, subgroup analysis based on treatment duration is not conducted here.

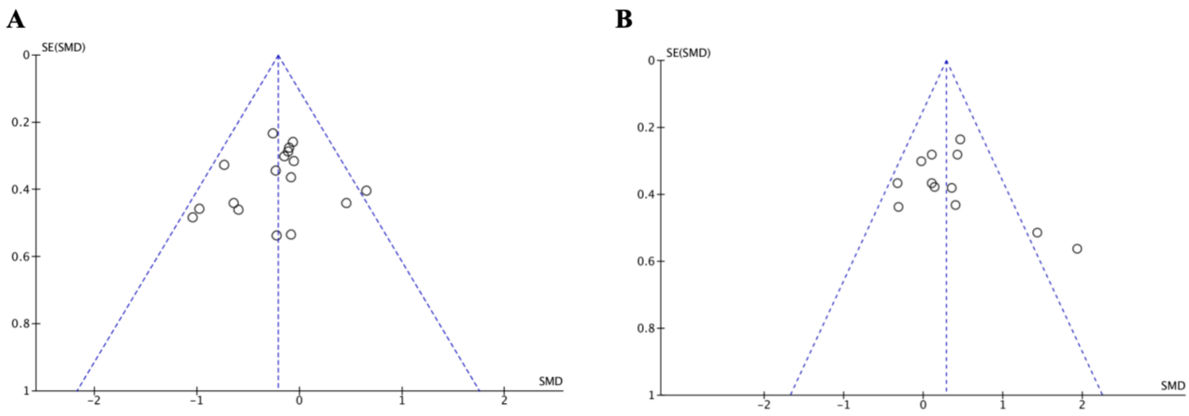

Figure S4 funnel plots of measures of (A) inhibition and (B) working memory in tDCS studies.
